# Do the issues of religious minority and coastal climate crisis increase the burden of chronic illness in Bangladesh?

**DOI:** 10.1186/s12889-022-12656-5

**Published:** 2022-02-10

**Authors:** Altaf Hossain, Md. Jahangir Alam, Janardhan Mydam, Mohammad Tareque

**Affiliations:** 1 Department of Statistics, Islamic University, Kushtia, 7003 Bangladesh; 2 Department of Statistics, University of Rajshahi, Rajshahi, 6205 Bangladesh; 3 Division of Neonatology, Department of Pediatrics, John H. Stroger, Jr. Hospital of Cook County, 1969 Ogden Avenue, Chicago, IL 60612 USA; 4 Department of Pediatrics, Rush Medical Center, Chicago, USA; 5 Bangladesh Institute of Governance and Management, Dhaka, Bangladesh

**Keywords:** Religious minority problem, Coastal climate crisis, Chronic illness, Disability, Distress financing, Social safety net, Bangladesh

## Abstract

**Background:**

Chronic illness with disability and its out-of-pocket expenditure (OOPE) remains a big financial challenge in Bangladesh. The purpose of this study was to explore how religious minority problem and coastal climate crisis with other common risk factors determined chronic illness with a disability and its financial burden in Bangladesh. Existing policy responses, especially, social safety net programs and their governance were analyzed for suggesting better policy options that avoid distress financing.

**Methods:**

Binary logistic and multiple linear regression models were respectively used to identify the factors of disability, and high OOPE based on Bangladesh Household Income and Expenditure Survey 2016 data.

**Results:**

We found that disable people had relatively higher OOPE than their non-disabled counterparts and this OOPE further surges when the number of disabilities increases. In addition to the common factors, the novelty of our findings indicated that the religious minority problem as well as the coastal climate crisis have bearing on the disability burden in Bangladesh. The likelihood of having a chronic illness with a disability was 13.2% higher for the religious minorities compared to the majorities (Odds ratio (OR): 1.132, 95% confidence interval (CI): 1.033–1.241) and it was 21.6% higher for the people who lived in the exposed coast than those who lived in the non-exposed area (OR: 1.216, 95% CI: 1.107–1.335). With disabilities, people from the exposed coast incurred higher OOPE than those from the non-exposed areas. Although receiving assistance from social safety net programs (SSNPs) seemed to reduce their high OOPE and financial distress such as selling assets and being indebted, the distribution was not equitably and efficiently managed to confirm the process of inclusion leakage-free. On average, those who enrolled from the minority group and the exposed coast paid the relatively higher bribes.

**Conclusions:**

To reduce burden, the government should strengthen and specify the existing SSNPs more for disable people, especially from the minority group and the exposed coast, and ensure the selection process more inclusive and leakage-free.

**Supplementary Information:**

The online version contains supplementary material available at 10.1186/s12889-022-12656-5.

## Introduction

### Background

The non-communicable diseases (NCDs)are the leading causes of death in low and middle-income countries (LMICs) [1–3]. One in three adults worldwide has multiple chronic conditions: cardiovascular diseases alongside diabetes, depression as well as cancer, or a combination of three, four, or even five or six diseases at the same time [4]. These NCD-related morbidities/disabilities and mortalities reduce labor productivity, increase healthcare expense and erode savings, which finally lead to having an adverse effect on economic growth and development [5]. In the last few decades, Bangladesh has made tremendous progress in primary healthcare by reducing fertility and, maternal and child mortality. However, new population and health challenges have emerged and been arising from rapid demographic and epidemiological transitions, urbanization, climate change, and the burdens of NCDs or chronic diseases. Not all the chronic illnesses always create economic/financial burdens until they are turned into any disability. Especially, some genetically inherited chronic illnesses can be carried out by individuals for a long time without showing any symptom or disability. However, chronic NCDs are the cause of disability in 68% of people worldwide and 84% in LMICs like Bangladesh [6]. So, we were concerned with chronic illness with disabilities to take into account their immediate financial burdens.

**Fig. 1:**
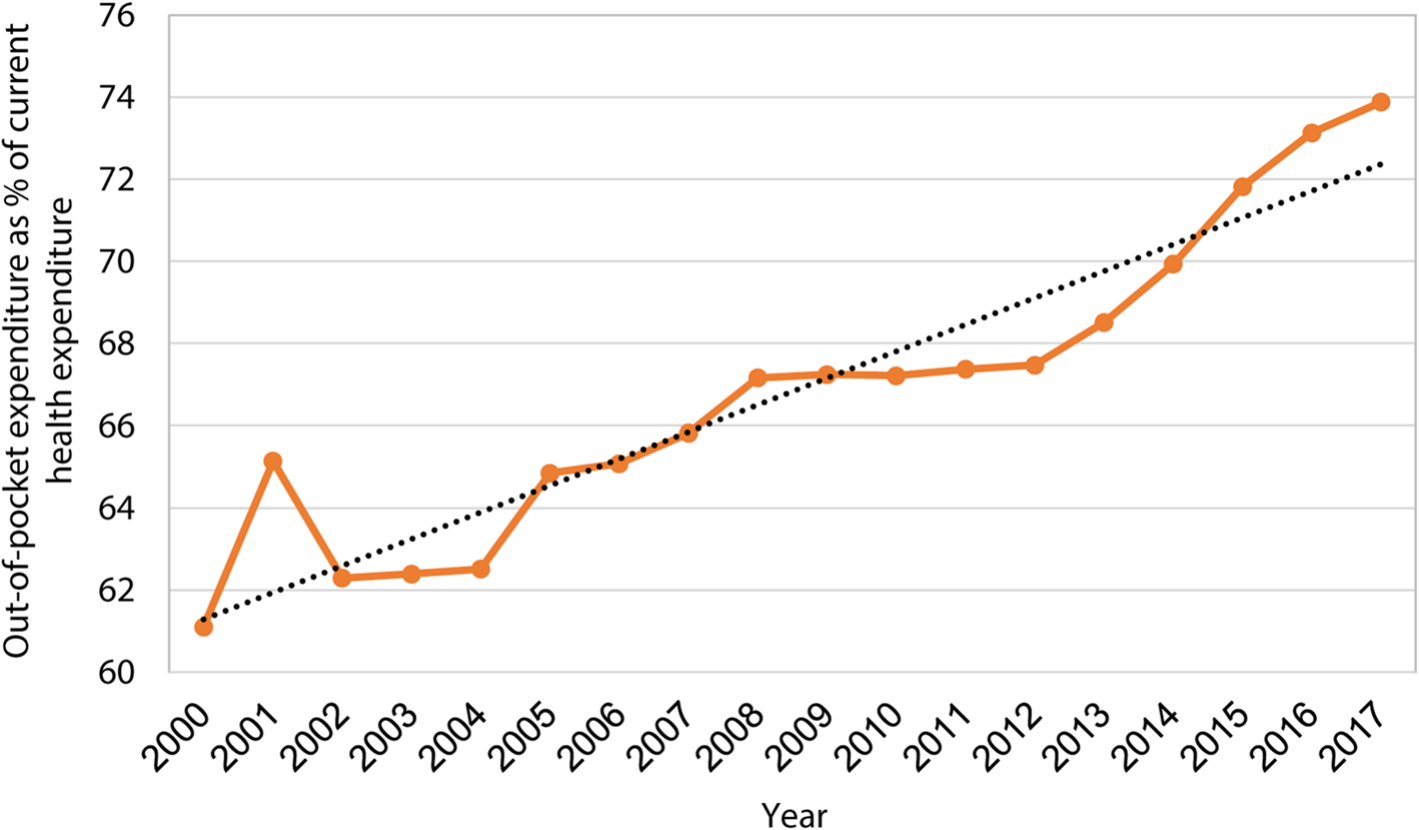
The trend of out-of-pocket expenditure as % of current health expenditure (CHE) in Bangladesh (Data Source: WHO)

Out-of-pocket expenditure (OOPE) is a measure of financial burden of chronic illness and its associated disabilities. According to WHO, out-of-pocket expenditures/payments are defined as direct payments made by individuals to healthcare providers at the time of service use, excluding any prepayment for health services in any form or net of any reimbursement by the health insurer (i.e., any reimbursements from health insurance schemes are deducted) [7, 8]. As a measure of financial burden, OOPE is higher for the patients with the number of chronic illnesses and disabilities [9]. This higher OOPE leads to income deficiencies, lack of healthcare access and the poor quality of life. The government of Bangladesh, in the national health policy (NHP) revised in 2011, set a goal to reduce the OOPE to 32% by 2032 [10]. Despite this goal, Bangladesh has emerged as one of the countries with the highest health burdens in the globe. Bangladeshi households are facing the burden of chronic diseases where OOPE remains the most substantial portion for healthcare, and their access to health insurance is rare [11]. Consequently, multiple chronic diseases and their several types of disabilities in Bangladesh have been gradually putting its OOPE on the dramatic rise as a percentage of health expenditure, from 67% in 2011 to almost 74% in 2017 (Fig. 1).

In many countries like Bangladesh, the poorest are at higher risk for chronic illness as they are least able to cope with the financial consequences [12]. As a result, they experience distress financing, which refers to borrowing money or selling assets to meet the OOPE on healthcare. About 150 million people suffered from distress financial in seeking healthcare services [13]. Some studies such as Sauerborn et al. [14], Onarheim et al. [15], Mishra and Mohanty [16], and Pal [17] suggest that households from LMICs consider common forms of distress financing such as borrowing from relatives and friends, loans from financial institutions and money lenders, selling assets, mortgaging assets, selling harvest crops and selling livestock.

### Research gap

In Bangladesh, a higher OOPE is observed to be linked with chronic disability, and the possibility of being disabled is higher among those individuals with a lower educational level, no (income) earning status, no health-seeking behavior, and those in the lowest wealth quintile [11]. In 2010, Molla et al. [18] also found that the presence of chronic disease increases OOPE, and it was higher among the urban dwellers and males in Bangladesh.

However, global climate change or its affected regions are found to be associated with higher NCD risk [19]. According to the Intergovernmental Panel on Climate Change (IPCC) report, Bangladesh being low-lying coastal region is one of the most vulnerable countries to climate change. It was evident that the prevalence of NCDs increases in the coastal region of Bangladesh [20–23]. Moreover, Price et al. [24] and Modesti et al. [25] reported higher NCD rates among the minority people in the US and Europe. Though the nature of the minority problem is somewhat different in Bangladesh, the violence and discrimination against religious minorities (non-Muslims) have also been continuously taking place for more than eight decades. Particularly, the Hindu population was targeted during the 1947 Partition and the bloody civil war in 1971. Consequently, minority population has declined to 9.6% today from 23.1% in 1971 [26], and Hindus were showing less happier than Muslims in Bangladesh [27].

These existing studies did not examine how ‘region’ (on climatic considerations) and ‘religion’ (as minority issue) increase the chronic illness with disability and financial burdens (OOPE and distress financing) in Bangladesh.

To reduce disability burden and avoid distress financing, the government of Bangladesh has already strengthened previous NCD programs as well as initiated some new types of safety net programs [28–31]. But without assessing their effectiveness, the existing studies like Molla et al. [18], traditionally recommended that government should offer relevant NCDs and safety net programs. Though Rahman and Choudhury [31] assessed some social safety net programs, they were limited to finding any occurrence of leakage such as paying an entry fee or bribe and fraudulent muster roll in the inclusion process of vulnerable people.

### Objectives

In our study, we made an attempt to take into account the issues of religious minority, coastal climate crisis, distress financing and the effectiveness of social safety net among the people with chronic illness in Bangladesh. Our main objective was to explore how religious minority problem and coastal climate crisis affect chronic illness with a disability and its financial burden of out-of-pocket expenditure in Bangladesh. To recommend better policy options, we contributed to check whether the social safety net programs tended to reduce out-of-pocket expenditure. We also examined the irregularities or leakages in the distribution of SSNPs by religion and region.

## Methods

### Data source

This study was designed based on secondary data from a nationally representative and unique survey, the Bangladesh Household Income and Expenditure Survey (HIES) 2016 (HIES2016). This survey was carried out by the Bangladesh Bureau of Statistics (BBS) from April 2016 to March 2017. The final report on this 16th round of HIES described the survey objectives, survey design, sampling technique, survey tools, measuring system, sample size calculation, quality control, and the questionnaire’s new modules [32]. The HIES2016 covered the ever highest number of 46,080 households selected from 2304 primary sampling units (PSUs), from 20 strata under 3 basic localities (rural, urban and city corporation): 8 rural, 8 urban, and 4 statistical metropolitan areas (Dhaka, Chattogram, Rajshahi and Khulna). It contains information on the household, disability, education, health, housing, and a wide range of socio-economic factors (e.g., family earnings, consumption and expenditures, assets, housing conditions, as well as data on demographic variables, education, employment) that has a strong role in the decision making process for the government.

The HIES 2016 followed a stratified two-stage cluster sampling design. At the first stage, PPS (probability proportional to size) systematic sampling technique was used to draw a total of 36 PSU’s from each district, where the number of households in each PSU being the measure of size. Enumeration Area, a cluster of around 110 households of Population Census 2011, was treated as PSU for this sample design. After selection of the PSU’s, a complete household listing in these selected PSU’s was done in the field. Thus, the total calculated sample size for the survey stands at 46,080 (2304 × 20) households. However, a total of 46,076 households were surveyed, with 32,096 from rural areas and 13,980 from urban areas. Among the selected households, a total of 186,076 individuals were interviewed, 130,435 from rural areas and the remaining 55,641 from urban areas. We considered both household and individual-level data in our analysis.

### Study data

For calculating the wealth quintile using household characteristics, we first converted each of the household characteristics into a binary variable. Based on the binary variables, we computed the wealth quintile using a multivariate technique principal component (PC) analysis, where the first PC score was considered as the wealth quintile. Then these quintile values were allocated to all individuals based on households.

**Fig. 2:**
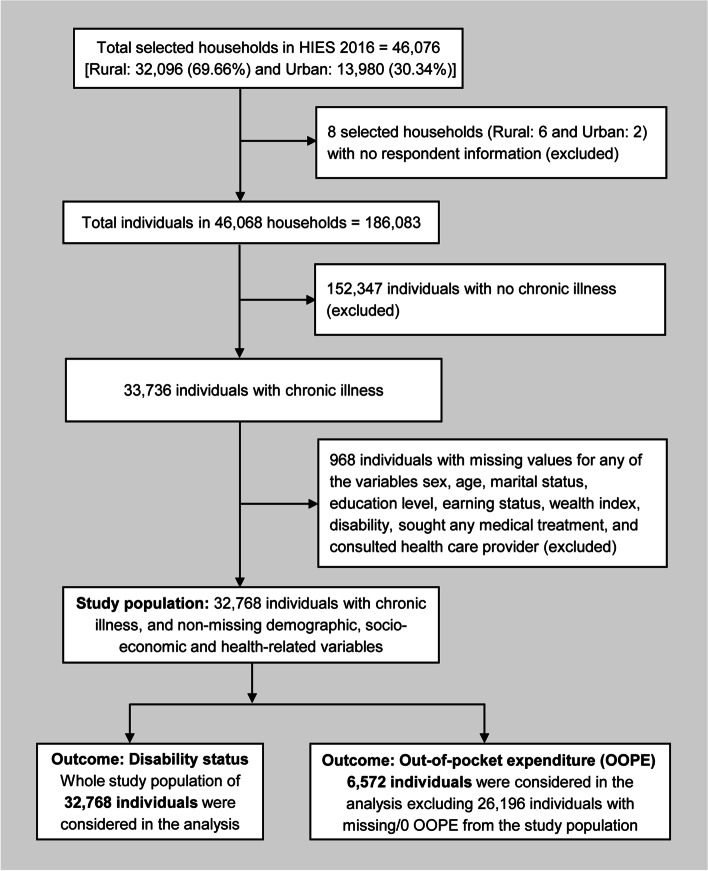
The diagram of the process of observation inclusion/exclusion

To finalize the study population for our study, we excluded some observations from the source data based on our inclusion/exclusion criteria (Fig. 2). Among 46,076 households, eight households were primarily excluded due to the lack of respondent’s information. Among the 186,083 individuals in the remaining 46,086 households, 152,347 were excluded as they did not suffer from chronic illness. Again, 968 individuals were excluded due to missing information on any of the socio-economic and demographic factors considered in our study. So far, we got 32,768 chronically ill individuals who constituted our Study Population to be analyzed in this study. For out-of-pocket expenditure analysis, data from 6572 chronically ill individuals were considered after excluding 26,164 individuals with missing OOPE (among them, 22,771 did not seek medical treatment and 3393 did not report OOPE) and 32 individuals with zero (0) OOPE from the study population of 32,768 individuals. We excluded zero values of OOPE from the analysis to avoid complexity in the normalization of data (e.g., the natural logarithm of zero is undefined). The whole process of inclusion/exclusion is depicted in the following Fig. 2.

### Study variables

#### Outcome variables

For conceptualizing disability, an International Classification of Functioning (ICF), Disability, and Health (ICFDH) was established by the World Health Organization (WHO) (https://www.who.int/classifications/icf/en/). Implementation of the ICF-based technique for disabilities requires the improvement of new measurement models to conduct both surveys and censuses. A useful small set of six disability-related questions was developed and adopted by the Washington Group for national censuses and surveys [33]. The HIES2016 utilized these six disability-related questions to be consistent with the ICFDH [33] and they are on difficulty, (i) for seeing even if he/she is wearing glasses, (ii) in hearing even if he/she is wearing a hearing aid, (iii) for walking or climbing or any other physical movement, (iv) in remembering or concentrating, (v) in self-care such as washing all over or dressing, feeding, toileting, etc., and (vi) in communicating. In the HIES2016, each individual of the household was asked to provide information about the presence of any disability and the severity of the disability. Each question had four response levels: (1) No Difficulty, (2) Yes, Some Difficulty, (3) Yes, Severe Difficulty or (4) Yes, Can’t see/hear/walk/remember/self-care/communicate at all. For the convenience of analysis, we converted the presence of disability into two groups: ‘0’ denoted for “No difficulty” and ‘1’ for “Any level of difficulty” [33–35]. Moreover, an outcome variable was defined as ‘chronic illness with a disability’ based on disability status and chronic illness (chronic fever, injuries/disability, chronic heart disease, respiratory disease/asthma/bronchitis, diarrhea/dysentery, gastric or ulcer, blood pressure, arthritis/rheumatism, skin problem, diabetes, cancer, kidney diseases, liver diseases, mental health, paralysis, ear/ENT problem, eye problem, and others) with two values: 1 denoting “Presence of at least one of the above six disabilities with chronic illness” and 0 denoting “Absence of disability with chronic illness”. For the second burden measurement, OOPE for healthcare was created by adding up direct medical costs, including hospital outpatient fees, medicine, admission or registration fees, physician fees, diagnostic fees, and any other associated medical supplies and direct non-medical costs, including transportation and conveyance, lodging, tips, and other associated costs [32]. Indirect costs such as loss of opportunity, productivity, and other intangible costs, including pain and suffering, were not captured in the HIES2016 by the BBS. Therefore, we were limited to include only the direct healthcare costs in the calculation of OOPE. In Bangladesh, OOPE is measured in Bangladeshi Taka (BDT) equivalent to the U.S. $0.01163 (i.e., 1 USD = 86 BDT).

#### Independent variables

**Fig. 3:**
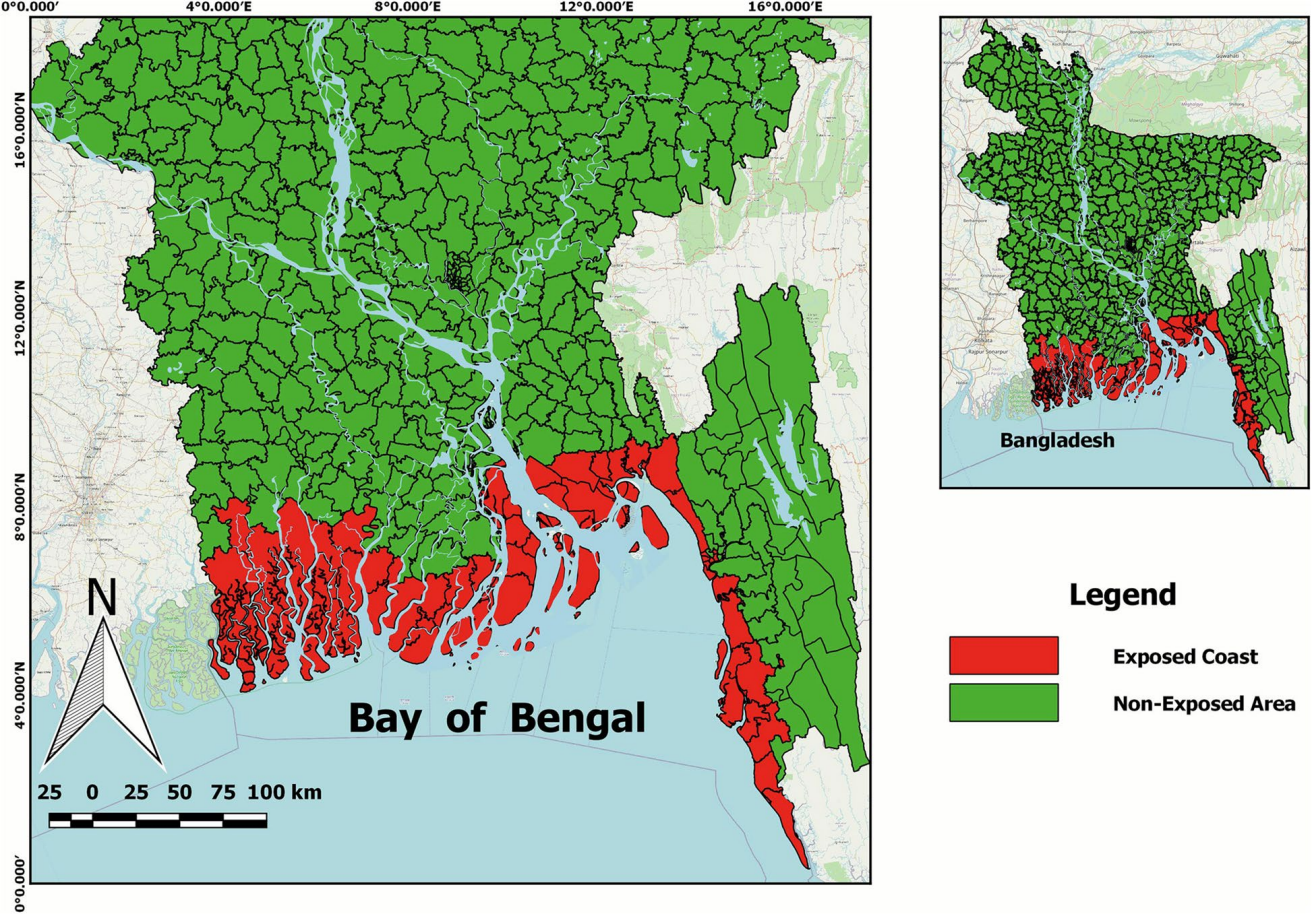
Coastal regions of Bangladesh. This figure has been created by a free QGIS (version 2.8.5-Wien) software (https://qgis.org/en/site/ or https://qgis.org/downloads/)

To take into account the minority problem and the coastal climate crisis, we defined and categorized ‘Religion’ as ‘Muslim/Non-Muslim’ and ‘Region’ as ‘Exposed Coast/Non-Exposed’. The ‘Non-Exposed’ region consists of interior coast and non-coastal areas. In Bangladesh, the Muslims are the majority and the non-Muslims (Hindus, Buddhis, Christians and others) led by Hindus are the minority. A detailed delineation of the exposed coast and non-exposed areas are provided in Uddin and Kaudstaal [36], and Bahauddin et al. [37]. The coastal regions of Bangladesh, with 19 districts containing 147 Upazilas, cover/occupy 32% of the country’s total geographic area, wherein 28% of the country’s total population live. To be more focused, we recognized a further division between the coastal areas as the exposed coast and the interior coast; where the former areas with 48 Upazilas are exposed to the sea and/or lower estuaries, and the later areas with the remaining 99 Upazilas located behind the exposed coastal were added to the non-exposed category in our study. So, the non-exposed areas consist of coastal interior and non-coastal areas. We depicted the exposed coast and non-exposed areas in Fig. 3 using freely available QGIS (version 2.8.5-Wien) software (https://qgis.org/en/site/ or https://qgis.org/downloads/).

The explanatory variables, religion and region by adjusting other control variables, demographic (such as age, sex, education, and marital status), healthcare provider (being categorized as public, private, pharmacy/dispensary, traditional, and other), wealth quintile for economic status (grouped into poorest: lowest 20%, poorer: 2nd quintile, middle: 3rd quintile, richer: 4th quintile, and richest: upper 20%), and employment/earning status (Yes/No) were used for predicting two burdens: chronic illness with a disability and OOP healthcare expenditure. Respondent’s age was categorized as childhood (≤ 19 years), young adulthood (20–39 years), middle-aged (40–64 years), senior-aged (65–84 years), and old senior-aged (≥ 85 years) [38]. We divided marital status into three groups: married, unmarried, and others (widowed, divorced, or separated). Likewise, Educational level was grouped as no education, primary, secondary, higher secondary, and higher education. The list of predictors of OOPE included one extra variable that describes whether respondents were currently enrolled or received any assistance from any SSNP.

### Statistical analysis

Data on the different variables were summarized using descriptive statistics. We also tabulated chronic illness by disability status in frequency count and percentage. Normality of the OOPE was checked using the Kolmogorov-Smirnov test before commencing the analyses of OOPE. Since the distribution of OOPE was non-normal (right-skewed), we summarized OOPE by the median and interquartile range (IQR), and the Wilcoxon-Mann-Whitney test was used to compare OOPE between two groups. We normalized the distribution of right-skewed OOPE data by applying a natural logarithmic transformation to OOPE. Bi-directionally connected two burdens – health and financial – were linked in box plots. Multiple logistic regression analysis was performed to evaluate the association of disability status with religion and region controlling for other explanatory variables, and to calculate odds ratios (ORs) for determining the odds of developing disability among minority group and people from the exposed coast compared to their respective counterparts. Multiple linear regression model was used with the normalized OOPE as the dependent variable to assess the influence of explanatory variables on OOPE. Data processing (Data cleaning, derivation of required variables and creating analysis dataset), validation, and all statistical analyses were performed using the SAS 9.4 software (SAS Institute, Inc., Cary, North Carolina, USA).

## Results

### Background characteristics of study population

Table 1 represents the descriptive statistics of the study variables. Among the study population, non-muslims were the minority group (11.50%) compared to the Muslims (88.50%). A significant portion (11%) of the study population lived in the exposed coast area of Bangladesh. A larger proportion of the study population were rural residences (70.40%), females (55.37%), middle-aged (46.44%), illiterates (45.43%), married (77.55%), non-earners (62.35%), healthcare non-seekers/non-receivers (69.49%). In the study population, 18.11% of the chronically ill people reported having at least one disability (single disability 9.85% and multiple disabilities 8.26%). We found a high prevalence of chronic gastric or ulcer (20.46%), arthritis/rheumatism (13.80%), chronic respiratory disease/asthma/bronchitis (10.23%), blood pressure (9.67%), and chronic heart disease (7.45%) among the chronically ill people (Table S1 of Supplementary file 1). All together, these highly prevalent chronic diseases, gastric/ulcer, arthritis/rheumatism, respiratory diseases or asthma or bronchitis, blood pressure and heart disease, contributed around 62% of total chronic illnesses. Among the respondents having chronic illness with at least one disability, the higher prevalence was observed for paralysis (58.61%), maternal health (49.23%), eye problem (41.28%), cancer (27.87%), and injuries/disability (25.34%).

**Table 1:** Background characteristics of study population^a^

Characteristic	Number of individuals (***n***)	Percentage (%)
**Demographic**		
**Religion**		
Muslim	29,001	88.50
Non-muslim	3767	11.50
**Coastal region**		
Coastal exposed	3597	10.98
Coastal interior and Non-coastal	29,171	89.02
**Gender**		
Male	14,624	44.63
Female	18,144	55.37
**Residence**		
Rural	23,070	70.40
Urban	9698	29.60
**Age group**		
Childhood (≤19 years)	2662	8.12
Young adult (20–39 years)	9497	28.98
Middle aged (40–64 years)	15,219	46.44
Senior aged (65–84 years)	4872	14.87
Old senior aged (≥85 years)	518	1.58
**Marital status**		
Married	25,413	77.55
Unmarried	3280	10.01
Others	4075	12.44
**Socio-economic**		
**Education level**		
No education	14,885	45.43
Primary	8125	24.80
Secondary	7665	23.39
Higher secondary	1109	3.38
Higher education	984	3.00
**Income earner**		
Yes	12,338	37.65
No	20,430	62.35
**Wealth quintile**		
Poorest	5530	16.88
Poorer	6380	19.47
Middle	7079	21.60
Richer	7222	22.04
Richest	6557	20.01
**Medical/Health**		
**Chronic illness with at least one disability**		
No disability	26,834	81.89
Any disability	5934	18.11
**Multiple disability status**		
No disability	26,834	81.89
Single disability	3228	9.85
Multiple disabilities	2706	8.26
**Sought medical treatment**		
Yes	9997	30. 51
No	22,771	69.49
**Treatment from healthcare provider**		
No treatment/consultancy	22,771	69.49
Public	1919	5.86
Private	5277	16.10
Pharmacy/Dispensary	2376	7.25
Traditional	298	0.91
Others	127	0.39

^a^ Study population consists of individuals with chronic illness, and non-missing demographic, socio-economic and health-related variables

### Bivariate distribution and association between disability status and risk factors

The associations of the selected risk factors with the disability status are shown in Table 2. We found that minority people had a significantly higher disability rate compared to the majority group (19.86% versus 17.88%, *P* = 0.003), which indicates that disability status was significantly associated with religion. Region was associated with disability status, exhibiting a significantly higher prevalence of disability among the people who lived in the exposed coast than those who lived in the non-exposed area (20.27% versus 17.84%, *P* < 0.001). Regarding remaining risk factors, the disability rate was significantly higher for those chronically ill people who were rural residences, senior-aged, married, illiterate or primary educated, unemployed and poorest or poorer, sought medical treatment, and received healthcare from public/private healthcare providers. In other words, residence, age group, marital status, education level, employment status, wealth quintile, healthcare seeking, and healthcare receiving were significantly associated with disability status of chronically ill people.

**Table 2:** Bivariate association of selected risk factors with disability status

Characteristic	Total	Chronic illness with		***P***-value
		At least one disability ***n*** (%)	No disability ***n*** (%)	
**Demographic**				
**Religion**				
Muslim	29,001	5186 (17.88)	23,815 (82.12)	0.0031
Non-muslim	3767	748 (19.86)	3019 (80.14)	
**Coastal region**				
Coastal exposed	3597	729 (20.27)	2868 (79.73)	0.0004
Non-exposed^a^	29,171	5205 (17.84)	23,966 (82.16)	
**Gender**				
Male	14,624	2652 (18.13)	11,972 (81.87)	0.9145
Female	18,144	3282 (18.09)	14,862 (81.91)	
**Residence**				
Rural	23,070	4389 (19.02)	18,681 (80.98)	< 0.0001
Urban	9698	1545 (15.93)	8153 (84.07)	
**Age group**				
Childhood (≤19 years)	2662	303 (11.38)	2359 (88.62)	< 0.0001
Young adult (20–39 years)	9497	655 (6.90)	8842 (93.10)	
Middle aged (40–64 years)	15,219	2714 (17.83)	12,505 (82.17)	
Senior aged (65–84 years)	4872	1934 (39.70)	2938 (60.30)	
Old senior aged (≥85 years)	518	328 (63.32)	190 (36.68)	
**Marital status**				
Married	25,413	3996 (15.72)	21,417 (84.28)	< 0.0001
Unmarried	3280	449 (13.69)	2831 (86.31)	
Others	4075	1489 (36.54)	2586 (63.46)	
**Socio-economic**				
**Education level**				
No education	14,885	3639 (24.45)	11,246 (75.55)	< 0.0001
Primary	8125	1173 (14.44)	6952 (85.56)	
Secondary	7665	880 (11.48)	6785 (88.52)	
Higher secondary	1109	129 (11.63)	980 (88.37)	
Higher education	984	113 (11.48)	871 (88.52)	
**Income earner**				
Yes	12,338	1590 (12.89)	10,748 (87.11)	< 0.0001
No	20,430	4344 (21.26)	16,086 (78.74)	
**Wealth quintile**				
Poorest	5530	1155 (20.89)	4375 (79.11)	< 0.0001
Poorer	6380	1189 (18.64)	5191 (81.36)	
Middle	7079	1261 (17.81)	5818 (82.19)	
Richer	7222	1262 (17.47)	5960 (82.53)	
Richest	6557	1067 (16.27)	5490 (83.73)	
**Medical/Health**				
**Sought medical treatment**				
Yes	9997	1960 (19.61)	8037 (80.39)	< 0.0001
No	22,771	3974 (17.45)	18,797 (82.55)	
**Treatment from healthcare provider**				
No treatment/consultancy	22,771	3974 (17.45)	18,797 (82.55)	< 0.0001
Public	1919	406 (21.16)	1513 (78.84)	
Private	5277	1046 (19.82)	4231 (80.18)	
Pharmacy/Dispensary	2376	427 (17.97)	1949 (82.03)	
Traditional	298	52 (17.45)	246 (82.55)	
Others	127	29 (22.83)	98 (77.17)	

^a^ Non-exposed area: Coastal interior and Non-coastal area

### Distribution of out-of-pocket expenditure by disability status and its risk factors

**Fig. 4:**
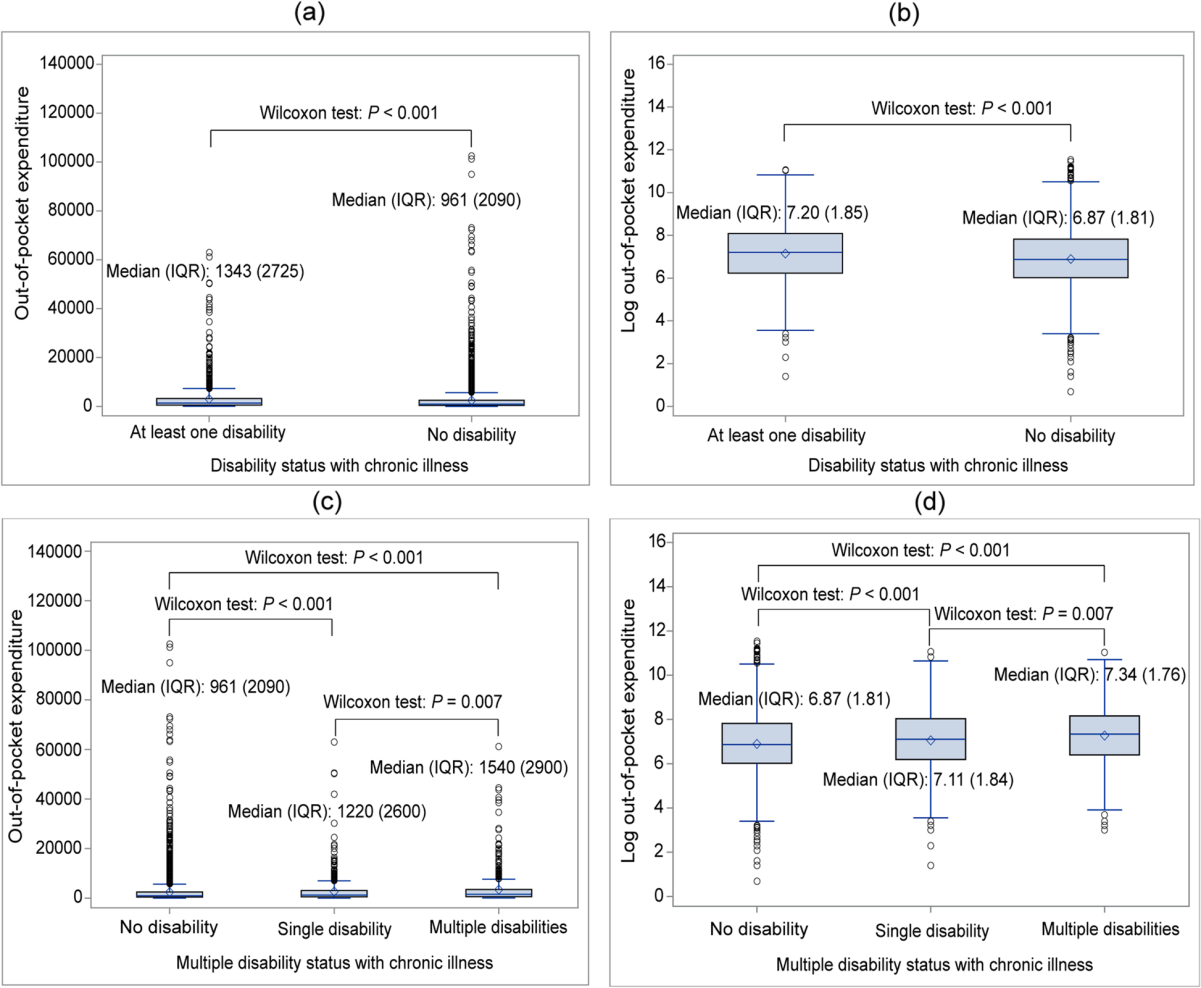
Box plots of out-of-pocket (OOP) expenditure (in BDT) and logarithmic OOP expenditure of chronically ill people. **a** Box plot of OOP expenditure stratified by disability status (at least one disability and no disability), **b** box plot of logarithmic OOP expenditure stratified by disability status (at least one disability and no disability), **c** box plot of OOP expenditure stratified by multiple disability status (no disability, single disability and multiple disability), and **d** box plot of logarithmic OOP expenditure stratified by multiple disability status (no disability, single disability and multiple disability)

Table 3 represents the distribution of OOP healthcare expenditure, in Bangladeshi taka (BDT), in the last 30 days by disability and its risk factors. Since the OOPE was non-normally distributed, we presented the descriptive summary statistics by median and IQR. The median OOP healthcare expenditure for chronic illness was BDT 1024 in the study population. Our study showed that the median OOPE was significantly higher for disabled people compared to their non-disabled counterparts (BDT 1343 versus BDT 961, *P* < 0.001) (Fig. 4 and Table 3). Referring to Fig. 4c, we found that the OOPE increased with the increase in the number of disabilities. The people with multiple disabilities had significantly higher OOPE than both the non-disabled (BDT 1540 versus BDT 961, *P* < 0.001) and the single disabled people (BDT 1540 versus BDT 1220, *P* = 0.007).

**Table 3:** Distribution of out-of-pocket payment by disability status and its risk factors

Components	Out-of-pocket payment BDT^**#**^ in last 30 days								
	Without disability			With disability			Overall OOPE		
	***n***	Median (IQR)	***P***-value	***n***	Median (IQR)	***P***-value	***n***	Median (IQR)	***P***-value
**Demographic**									
**Religion**									
Muslim	4730	995 (2080)	0.064	1185	1320 (2680)	0.403	5915	1030 (2214)	0.237
Non-muslim	512	850 (1828)		145	1500 (3250)		657	990 (2130)	
**Coastal region**									
Coastal exposed	691	1150 (2355)	< 0.001	213	1600 (2625)	0.010	904	1280 (2470)	< 0.001
Non-exposed^a^	4551	922 (2080)		1117	1250 (2700)		5668	1000 (2200)	
**Gender**									
Male	2190	940 (2030)	0.484	580	1400 (3400)	0.083	2770	1040 (2250)	0.860
Female	3052	974 (2100)		750	1250 (2485)		3802	1018 (2170)	
**Residence**									
Rural	3669	900 (2000)	< 0.001	1031	1245 (2678)	0.012	4700	990 (2120)	< 0.001
Urban	1573	1065 (2250)		299	1670 (2990)		1872	1140 (2430)	
**Age group**									
Childhood	483	730 (1940)	< 0.001	73	860 (1780)	0.021	556	785 (1890)	< 0.001
Young adult	1795	900 (1845)		172	1000 (2050)		1967	900 (1840)	
Middle aged	2403	1022 (2320)		616	1400 (2850)		3019	1080 (2540)	
Senior aged	535	1000 (2065)		405	1500 (2730)		940	1170 (2422)	
Old senior aged	26	2195 (4720)		64	1440 (2150)		90	1580 (2910)	
**Marital status**									
Married	4166	1000 (2108)	0.007	924	1356 (2780)	0.038	5090	1046 (2260)	0.003
Unmarried	553	820 (2050)		94	895 (1978)		647	833 (2050)	
Others	523	900 (1740)		312	1350 (2630)		835	1000 (2020)	
**Socio-economic**									
**Education level**									
No education	2143	850 (1730)	< 0.001	772	1070 (2230)	< 0.001	2915	900 (1860)	< 0.001
Primary	1446	921 (2020)		307	1600 (2950)		1753	1000 (2150)	
Secondary	1350	1150 (2540)		208	1920 (3310)		1558	1239 (2630)	
Higher secondary	171	1100 (2930)		23	1560 (3175)		194	1200 (2970)	
Higher education	132	1500 (3420)		20	5980 (6925)		152	1650 (4700)	
**Income earner**									
Yes	2039	870 (1790)	< 0.001	361	1150 (2500)	0.027	2400	900 (1867)	< 0.001
No	3203	1000 (2275)		969	1395 (2748)		4172	1080 (2440)	
**Economic status**									
Poorest	793	815 (1773)	< 0.001	260	950 (2200)	< 0.001	1053	850 (1800)	< 0.001
Poorer	1049	840 (1700)		283	1150 (2358)		1332	890 (1835)	
Middle	1145	850 (2020)		259	1800 (3410)		1404	955 (2220)	
Richer	1274	1000 (2070)		309	1315 (2290)		1583	1030 (2150)	
Richest	981	1285 (2714)		219	1946 (2990)		1200	1410 (2900)	
**Medical/Health**									
**Healthcare provider**									
Public	852	1400 (2505)	< 0.001	240	1400 (2400)	< 0.001	1092	1400 (2500)	< 0.001
Private	2916	1280 (2755)		764	1810 (3100)		3680	1400 (2850)	
Pharmacy/Dispensary	1263	500 (750)		279	680 (1080)		1542	550 (790)	
Traditional	156	565 (880)		32	565 (2488)		188	565 (890)	
Other	55	500 (1930)		15	295 (933)		70	420 (1330)	
Total	5242	961 (2090)		1330	1343 (2725)		6572	1023 (2202)	

*BDT* Bangladeshi Taka, *OOPE* Out-of-pocket expenditure, *IQR* interquartile range

^#^ 1 USD = 86 BDT

^a^ Non-exposed area: Coastal interior and Non-coastal area

The OOPE was higher for disabled minority people (BDT 1500) compared to the majority (BDT 1320). According to the residence setting, people who lived in the exposed coastal areas spent more OOP money than those who lived in the non-exposed area (BDT 1280 versus BDT 1000, *P* < 0.001). Also, the disabled persons from the exposed coast had higher OOPE than those who lived in the non-exposed area (BDT 1600 versus BDT 1250, *P* = 0.010). The OOPE exhibited a similar pattern for chronically ill non-disabled people (coastal exposed: BDT 1150 versus non-exposed: BDT 922, *P* < 0.001). Regarding other risk factors, OOPE was reported significantly higher among the people who were urban residences, senior-aged or old senior-aged, married, higher educated, unemployed, richest, and public/private healthcare receivers. We also noticed that OOPE was higher among disabled people than their non-disabled counterparts across all risk factors, indicating that disability increases the financial burden among chronically ill people.

### Distress financing

Table 4 represents the distribution of the source of financing for the treatment of chronically ill people with or without disabilities. In our study population, only 9% of chronically ill people responded to the sources of their treatment costs. The study found that most of the chronically ill respondents managed their treatment expenditure from their regular income (33.11%) and household saving (26.86%), and by borrowing money from friends/relatives/office/moneylender (14.19%). The proportion of people who sold livestock (disabled: 7.64% versus non-disabled: 6.70%) and permanent assets (disabled: 2.78% versus non-disabled: 1.56%), mortgaged their assets/land (disabled: 3.47% versus non-disabled: 2.46%), and borrowed from friends/relatives/office/moneylenders (disabled: 17.36% versus non-disabled: 13.17%) was higher for the chronic illness with disability than the chronic illness without a disability. Considering minority issue, the proportion of minorities was higher for the following sources of financing for treatment: regular income (minority: 35.29% versus majority: 32.97%), household saving (minority: 29.41% versus majority: 26.70%), selling livestock (minority: 8.82% versus majority: 6.81%), and assistance from friends and relatives (minority: 11.76% versus majority: 9.32%). Interestingly, the rate of borrowing money was less among minorities compared to the majority (minority: 8.82% versus majority: 14.51%), and the rate of mortgaging assets/land and selling personal belonging and permanent assets was zero. It was also found that the rates of selling personal belonging (exposed: 2.60% versus non-exposed: 0.19%) and agricultural product/tree (exposed: 6.49% versus non-exposed: 2.33%), and borrowing money (exposed: 16.89% versus non-exposed: 13.79%) was higher in the exposed coastal areas.

**Table 4:** Distribution of the sources of financing for the treatment

Financing treatment^**a**^		Chronic illness with		Religion		Region	
	Overall ***n*** (%)	At least one disability ***n*** (%)	No disability ***n*** (%)	Muslim ***n*** (%)	Non- muslim ***n*** (%)	Exposed coastal ***n*** (%)	Non- exposed ***n*** (%)
Regular income	196 (33.11)	45 (31.25)	151 (33.71)	184 (32.97)	12 (35.29)	25 (32.47)	171 (33.20)
Household saving	159 (26.86)	34 (23.61)	125 (27.90)	149 (26.70)	10 (29.41)	20 (25.97)	139 (26.99)
Sold personal belonging	3 (0.51)	2 (1.39)	1 (0.22)	3 (0.54)	0 (0.00)	2 (2.60)	1 (0.19)
Sold Livestock	41 (6.93)	11 (7.64)	30 (6.70)	38 (6.81)	3 (8.82)	3 (3.90)	38 (7.38)
Sold Agricultural product/Tree	17 (2.87)	4 (2.78)	13 (2.90)	16 (2.87)	1 (2.94)	5 (6.49)	12 (2.33)
Sold permanent assets	11 (1.86)	4 (2.78)	7 (1.56)	11 (1.97)	0 (0.00)	1 (1.30)	10 (1.94)
Mortgage of Assets/Land	16 (2.70)	5 (3.47)	11 (2.46)	16 (2.87)	0 (0.00)	2 (2.60)	14 (2.72)
Borrowed^b^	84 (14.19)	25 (17.36)	59 (13.17)	81 (14.51)	3 (8.82)	13 (16.89)	71 (13.79)
Assistance^c^	56 (9.46)	12 (8.33)	44 (9.82)	52 (9.32)	4 (11.76)	6 (7.79)	50 (9.71)
Other (specify)	9 (1.52)	2 (1.39)	7 (1.56)	8 (1.43)	1 (2.94)	0 (0.00)	9 (1.75)

^a^ A 5980 of respondents were missing/had no responses

^b^ Borrowed from friends/relatives/office/moneylender

^c^ Assistance from friends and relatives

### Social safety net programs (SSNPs)

Our study identified that almost 11% of the study population were enrolled or received any assistant from any SSNP (Table S2 of Supplementary file 1). Among the people who were enrolled in the SSNP, the higher proportion of enrollment was reported for old age allowance (36.17%), vulnerable group feeding (13.03%), gratuitous relief - food/cash (10.77%) and widow/deserted/destitute women allowances (8.73%). The majority of the enrolled people (82.71%) knew about these programs before participation, and most of them were selected by the selection committees or someone’s references. In our analysis (Table 5), it was evident that the rate of enrollment in or receiving assistance from any SSNP was higher among the disabled respondents (disabled: 18.66% versus non-disabled: 9.06%). Only 13.65% of the enrolled participants were selected in the SSNPs through a proper application by filling out the application form (Table S2 of Supplementary file 1). On the other hand, the rest of the enrolled participants (86.35%) were selected in the SSNPs through the committee and other special references. As a result, about 8% of the SSNP participants (10.12% of disabled and 6.83% of the non-disabled people) had to pay money to be selected in the SSNPs. On average they had to pay BDT 1335 where a disabled participant paid BDT 1298 and a non-disabled bribed BDT 1360 (Table 5). On average, the minority people had to pay a higher amount (minority: BDT 1500 versus majority: BDT 1313) and the people who lived in the exposed coast areas also paid a higher amount than those who lived in the non-exposed areas (BDT 1761 versus BDT 1304).

**Table 5:** Information about the payment of money (in BDT^a^) to be included in the social safety net program (SSNP)

Variable	Overall		Chronic illness with	
	N	Mean (SD)	At least one disability Mean (SD)	No disability Mean (SD)
**How much did you had to pay?**	278	1335 (1224)	1298 (1207)	1360 (1238)
**Religion**				
Muslim	245	1313 (1198)	1303 (1154)	1320 (1233)
Non-muslim	33	1500 (1408)	1259 (1686)	1621 (1274)
**Coastal area**				
Exposed Coastal	19	1761 (1480)	1050 (807)	2088 (1626)
Coastal interior and Non-coastal	259	1304 (1200)	1312 (1226)	1298 (1186)
**Residence**				
Rural	241	1387 (1221)	1353 (1231)	1410 (1218)
Urban	37	995 (1204)	914 (970)	1044 (1345)

^a^ 1 USD = 86 BDT

### Regressions for disability and out-of-pocket payment

In Table 6, our study summarizes the results of the multiple logistics regression (Model I) and multiple linear regression (Model II) analyses. Model I provides the odds of occurring at least one disability given the risk factors of our interest, and Model II estimates the regression coefficients of these risk factors for predicting the financial burden, OOPE. It was observed that the chronically ill minority (non-Muslims) had 13% higher odds of having a disability than the majority (Odds ratio [OR]: 1.132, 95% confidence interval [CI]: 1.033–1.241). The people who lived in the exposed coast had 22% higher odds to have a chronic illness with a disability than those in the non-exposed areas (OR: 1.216, 95% CI: 1.107–1.335). About other adjusted risk factors, the odds of chronic illness with disability was significantly higher among the persons who were males OR: 1.63, 95% CI: 1.499–1.776), rural residences (OR: 1.163, 95% CI: 1.078–1.254), middle-aged (OR: 2.719, 95% CI: 2.469–2.995), senior-aged (OR: 6.053, 95% CI: 5.414–6.768), old senior-aged (OR: 12.77, 95% CI: 10.365–15.732), unmarried (OR: 2.413, 95% CI: 1.993–2.921), illiterate (OR: 1.622, 95% CI: 1.307–2.014), primary educated (OR: 1.249, 95% CI: 1.003–1.555), and unemployed (OR: 2.036, 95% CI: 1.867–2.220).The multiple linear regression analysis (Model II) accordingly revealed that OOP healthcare expenditure was higher in the exposed coast. More specifically, the average log(OOPE) of the people who lived in the exposed coast was significantly higher by 0.278 than that of those who lived in the non-exposed areas (regression coefficient: 0.278, 95% CI: 0.187–0.368, *P* < 0.001). For the remaining risk factors, OOPE was significantly higher for the males, urban residences, higher-aged, unmarried, higher educated, unemployed, belonging to the highest wealth quintile, private healthcare receivers, and non-receivers of any assistance from any SSNP.

**Table 6:** Binary logistic and linear regressions for disability and out-of-pocket payment (in BDT^#^)

Variable	Model 1 Outcome: Disability status with chronic illness		Model 2 Outcome: Out-of-pocket expenditure	
	OR	95% CI	Estimate	95% CI
**Religion**				
Non-muslim vs Muslim	1.132**	(1.033,1.241)	−0.019	(−0.122, 0.085)
**Coastal region**				
Coastal exposed vs non-exposed^a^	1.216***	(1.107,1.335)	0.278***	(0.187,0.368)
**Gender**				
Male vs Female	1.633***	(1.500,1.777)	0.174***	(0.085,0.264)
**Residence**				
Rural vs Urban	1.160***	(1.075,1.251)	−0.082*	(− 0.158,-0.005)
**Age group**^**b**^				
Childhood vs Young adult	0.601***	(0.484,0.747)	− 0.375***	(− 0.590,-0.159)
Middle aged vs Young adult	2.718***	(2.468,2.994)	0.257***	(0.180,0.334)
Senior aged vs Young adult	6.040***	(5.401,6.754)	0.369***	(0.255,0.483)
Old senior aged vs Young adult	12.702***	(10.309,15.650)	0.514***	(0.234,0.793)
Marital status				
Unmarried vs Married	2.401***	(1.983,2.908)	0.124	(−0.080,0.328)
Others vs Married	1.754***	(1.609,1.913)	−0.067	(−0.172,0.038)
**Education level**				
No education vs Higher education	1.622***	(1.307,2.014)	−0.591***	(−0.807,-0.375)
Primary vs Higher education	1.249*	(1.003,1.555)	−0.4058***	(−0.622,-0.190)
Secondary vs Higher education	1.097	(0.881,1.367)	−0.258*	(−0.474,-0.042)
Higher secondary vs Higher education	1.074	(0.812,1.420)	−0.315*	(−0.585,-0.044)
**Income earner**				
No vs Yes	2.039***	(1.870,2.224)	0.294***	(0.205,0.384)
**Wealth quintile**				
Lowest 20% vs Upper 20%	1.012	(0.906,1.131)	−0.170**	(−0.282,-0.059)
2nd 20% vs Upper 20%	0.898	(0.806,1.000)	−0.124*	(−0.232,-0.015)
3rd 20% vs Upper 20%	0.888*	(0.799,0.986)	−0.169**	(−0.270,-0.068)
4th 20% vs Upper 20%	0.895*	(0.809,0.991)	−0.201***	(−0.319,-0.083)
**Treatment from healthcare provider**				
No treatment/consultancy vs Private	0.829***	(0.764,0.899)		
Public vs Private	1.052	(0.917,1.208)	−0.001	(−0.088,0.085)
Pharmacy/Dispensary vs Private	0.906	(0.793,1.035)	−0.925***	(−1.002,-0.849)
Traditional vs Private	0.935	(0.673,1.299)	−0.8017***	(−0.989,-0.615)
Others vs Private	1.099	(0.699,1.730)	−1.099***	(−1.400,-0.797)
**Currently enrolled / has received any assistance from any SSNP**				
Yes vs No			−0.201***	(−0.298,-0.103)

*, ** and *** indicate *P* < 0.05, *P* < 0.01, *P* < 0.001, respectively

^#^ 1 USD = 86 BDT

Abbreviation: *OR* Odds ratio, *CI* Confidence interval, *SSNP* Social safety net program

^a^ Non-exposed area: Coastal interior and Non-coastal area

^b^ Age groups: Childhood (<=19 years), Young adult (20–39 years), Middle aged (40–64 years), Senior aged (65–84 years) and Old senior aged (> = 85 years)

**Fig. 5:**
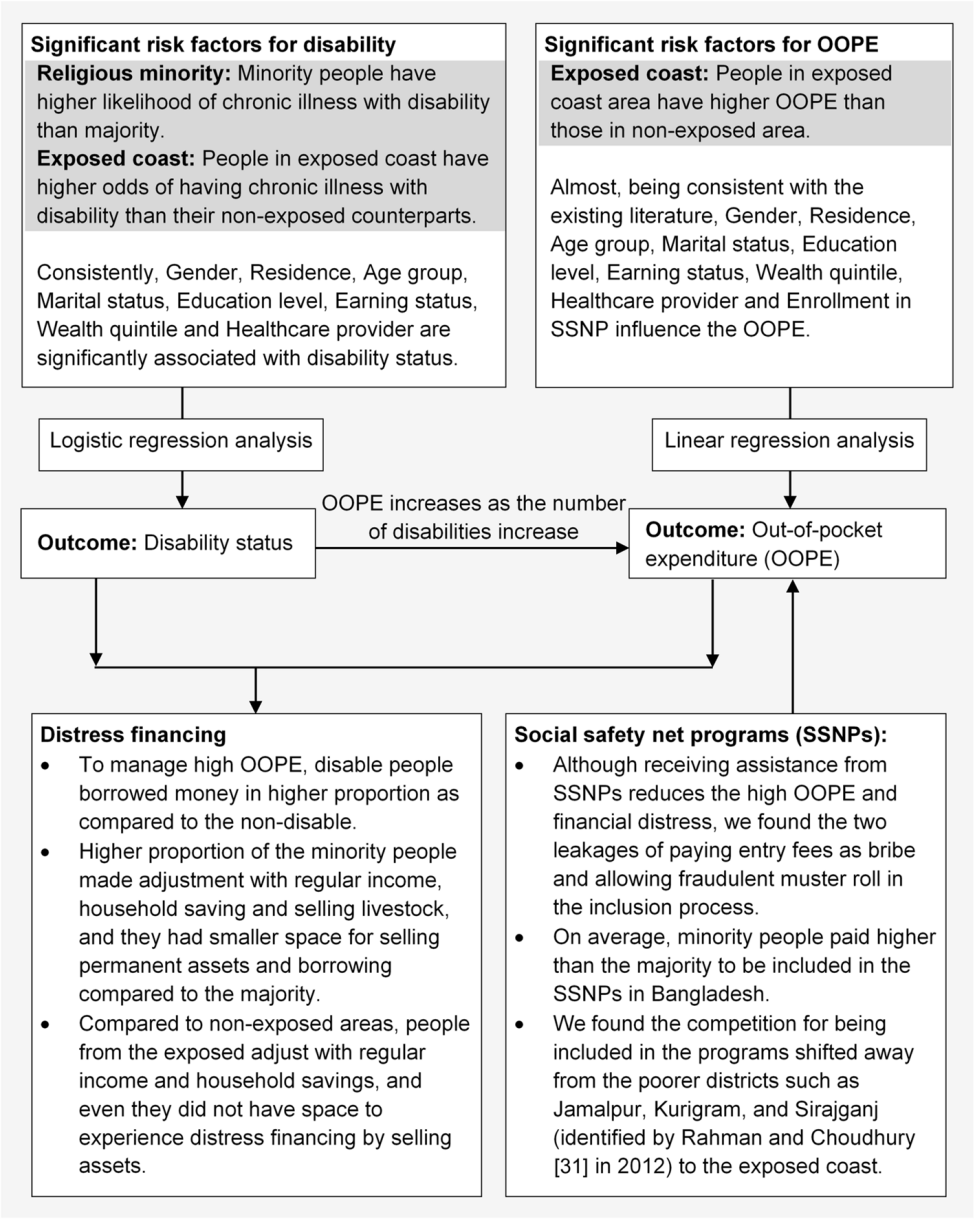
The diagram of the results of finding risk factors to disability burdens, distress financing and the assessment of SSNPs for reducing burdens

In Fig. 5, we have summarized our findings on (i) how the religious minority, the coastal climate crisis and posed special threats to chronic illness and financial burdens, (ii) how multiple disabilities increase financial burdens, (iii) distress financing stratified by disability status, religion and region, and (iv) the assessment of SSNPs for reducing these burdens.

## Discussion

This study, based on nationally representative HIES 2016 data, investigated the association of religion and region with chronic illness and its financial burden (OOPE) to examine if religious minority and coastal climate crisis significantly increase the burdens of chronic illness (i.e., disability and OOPE) in Bangladesh. Chronic illnesses such as cancer, heart diseases, liver diseases, diabetes, paralysis, etc., are increasing globally with associated comorbidities that warn a rising concern for assessing the disease-related burdens and efficiency of the healthcare system [39]. Therefore, the study also explored the relationship between chronic illness with/without any disability and OOPE and inspected how the multiplicity of disability increases the financial burden as OOPE. A significant prevalence of chronic illness was reported in our study, where 18% of the study population were suffering from an associated disability. The existing studies also reported that chronic illness conferred a heightened risk of disability [40, 41]. Since having a chronic disease does not necessarily imply to cause disability and financial burden, we focused on “chronic disease with a disability” to take into account the final burdens. In the study population, chronic gastric or ulcer, arthritis/rheumatism, chronic respiratory diseases, blood pressure and chronic heart disease were highly prevalent and contributed about 54% of total chronic diseases. Some previous studies claimed similar findings in Bangladesh [6, 42, 43]. We also found that paralysis exhibited the highest prevalence of disability among all chronic diseases as reported in Sultana et al. [11].

Our study identified the religion as a significant risk factor for disability; that is, the minority people had higher prevalence and odds of disability than the majority, which is consistent with the results reported by BBS [44] and Sultana and Gulshan [45]. In a study in India, Sarkar and Mahesh [46] viewed a similar finding in terms of a minority religion. Price et al. [24] and Modesti et al. [25] also confirmed the connection of minority problem to the rise of NCDs burdens in the US and Europe. The former group found that racial/ethnic minorities were 1.5 to 2.0 times more likely than whites to have most of the major NCDs in the US [24], and the later research group argued that the minorities originating from South Asia and Africa were found to have a higher risk of these diseases than native Europeans [25].

We observed a significantly higher disability rate and higher odds of incurring disability in the exposed coast compared to the non-exposed regions. The evidence suggested that the people living in these exposed areas are poor, and they get fewer healthcare facilities in Bangladesh [47]. The heat waves were associated with heart stroke, dehydration, and aggravation of cardiovascular diseases in elderly people [21], and salinity intrusion caused a high incidence of hypertension in the coastal areas [22]. Scheelbeek et al. [23] identified that increased sodium concentrations in drinking water in coastal Bangladesh caused high blood pressures among non-pregnant adults. Harris et al. [20], Rahman [21], MoEF [22] and Scheelbeek et al. [23] also explained higher NCDs in the coastal region in Bangladesh and other countries. As we found, a study in Ecuador reported that living in the coastal areas was associated with the higher risks of self-reported chronic illness [48].

On economic burden, the studies [49, 50] reported the ongoing epidemiologic transition of chronic illness and found its association with higher OOPE in some Asian countries. Being consistent with the findings by Sultana et al. [11], our study revealed that disabled people had significantly higher average OOPE than the non-disabled people. We also found that multiple disabilities increased the burden of OOPE compared to a single disability, as evident in Paez et al. [51] and Payne et al. [52]. Like the burden of experiencing a disability, the multiple linear regression analysis of OOPE also confirmed its higher financial burdens of disability in the disadvantaged groups with a few exceptions. We observed higher OOPE in the exposed coast compared to non-exposed areas. The reason for higher OOPE in the exposed coast might be their higher prevalence of chronic illness among the people. Though there is a dearth of researches, a study conducted in the United States reported an increasing trend in OOP healthcare expenditure in the coastal area of California [53]. However, we did not find any evidence of a significant difference in OOPE between the religious majority and minority.

### Distress financing

As a secondary objective, we explored the distress financing of the study population of chronically ill people. As a result of the higher financial burdens of disability, chronically ill people experienced distress financing by selling their assets or mortgage their assets/land or borrowing money to manage the higher OOPE on healthcare. After regular income and saving, borrowing is the most common strategy for coping with the economic or financial burden to meet higher healthcare costs in the poorer countries where social healthcare protection is limited. In our study, we found that, besides regular income and household saving, most of the chronically ill respondents managed their treatment expenditure by borrowing money from friends/relatives/office/moneylender. Datta et al. [54] also linked between NCDs and the adverse economic effects on the households, and observed the potential displacement effect that might happen through the high medical expense and lower spending on essentials in Bangladesh. Unfortunately, a higher rate of distress financing was found among the religious minority group by adjusting with their regular income, household savings, assistance from friends and relatives, and selling livestock. Since the minority people had long been evicted from their own land and did not save earnings assets, they were not able to manage financing for treatment from other sources. The people of exposed coast region experienced more distress financing by selling personal belonging and agricultural product/tree, and borrowing money than the people from non-exposed areas.

### Social safety net programs (SSNPs)

To reduce the growing burden of NCDs and its distress financing, the World Health Organization (WHO) has set its 2013–2020 Action Plan for the Global Strategy for the Prevention and Control of chronic diseases [55]. Almost at the same time, the government of Bangladesh has made a National Health Strategy 2012–2032 that synchronizes a goal to realize the universal health coverage (UHC) by the year 2032. Though Bleich et al. [43] and Biswas et al. [29] concluded that government of Bangladesh successfully initiated a good number of policy responses towards the global action plan, these NCDs programs are still in their infancy stage and lack rigorous monitoring and evaluation. However, these NCD programs do not provide any assistance directly through cash or food transfers, the SSNPs in Bangladesh were designed to reduce poverty and protect vulnerable/disadvantaged people through different forms of cash and food aids. Over the time, SSNPs have graduated to a mainstream social and development concern and committed to ensuring Decent Work and Economic Growth for all (SDG8) and reduce inequalities (SDG10) [28]. In our study, almost 11% of the study population were enrolled or they received any assistant form any SSNP (Table S2 of Supplementary file 1). The higher proportion of enrollment was found for old age allowance, vulnerable group feeding, gratuitous relief - food/cash and widow/deserted/destitute women allowances. Though old age allowance did not directly serve the chronically ill and disabled people, the program had more space to include these people as the old age people were more likely to experience chronic illness with disability. However, a government study stated that SSNPs in Bangladesh cover disability benefits for different age groups in different forms [56]. Under the social protection schemes, now it is claimed that the total number of insolvent disable stood at 10 lakh receiving BDT 700 a month in 2018, and from 2019 to 20 both figures are assumed to be increased by “promotion approach” and “protection approach” [28]. In our analysis, the majority of the people involved in the SSNPs knew about these programs before participation, and most of them were selected by the selection committees or someone’s references.

In our results, only a very small portion of the enrolled participants were selected in the SSNPs through a proper application by filling out the application form. As a result, some of the SSNP participants had to pay money to be selected in the programs. Disabled participants had to pay lower than non-disabled people. Discrimination exists not only in the distribution of advantages or social transfers, but it also lies in the process of taking or offering bribes. Although, SSNPs are motivated by both equity and efficiency considerations, and then ensure economic growth by reducing inequality [30], a PPRC-UNDP Research Initiative [31] found some ways of worrying leakage in the inclusion process of poor and vulnerable people. They identified two dominant leakage allegations such as having to pay an entry fee (as a bribe) in cases of allowance programs, and leakage through fraudulent muster roll (connecting to local government and political party). On the other hand, our regression model identified that receiving any assistance from any SSNP seemed to reduce the high OOPE (Table 6) and financial distress, but the distribution was not efficient and equitable in Bangladesh (Table S2 of Supplementary file 1). Like Rahman and Choudhury [31], we also observed that the SSNPs were unevenly managed and seemed to have the two leakages of paying entry fees as a bribe and allowing fraudulent muster roll in the process of inclusion. In addition, we examined the distribution of leakages by religion and region. The minority and the people from the exposed coast (southern part of Bangladesh) had to pay higher amount in bribes. But Rahman and Choudhury [31] in 2012 found that the entry fee burden was much more pronounced in the poorer districts (e.g., Jamalpur, Kurigram, and Sirajganj) of northern part of Bangladesh, where there was a more intense competition among the poor for the limited allowance card available. Therefore, it seemed to us that the intense completion for the limited available allowances was shifted away from the poorer northern districts to the southern districts of the exposed coast.

## Conclusions

As NCD, chronic illness with disability has a greater impact on financial burden in OOPE, in turn, it leads to an overwhelming financial distress in Bangladesh. In this study, we contributed to examine how the minority problem and coastal climate crisis increase the disability and financial burden further. We also checked how and whether the existing SSNPs were managed to reduce these burdens in the country. A higher prevalence of disability was observed in the minority people. Both disability and OPPE were significantly higher in the coastal area of Bangladesh. As a policy response, the SSNPs appeared to reduce higher OOPE and distress financing, but the distribution of SSNPs was not seemed to be efficient and equitable, even by religion and region. In the inclusion process of SSNPs, the minority and people from the coastal area had to bribe higher amount. Along with emphasizing on other risk factors or vulnerable people, more attention needs to be directed toward the exposed coast and the minority population with associated disabilities in Bangladesh. Therefore, the government should efficiently strengthen and specify the existing SSNPs for disabled people by ensuring the process of inclusion free and fair, and equitable in every aspect. In this regard, the government of Bangladesh can increase the size, beneficiaries and specialization of the social safety net programs. The selection process of the poor people in the programs should be more inclusive by including local management and local opinion leaders, e.g., NGOs and local school or college teachers.

## Supplementary Information

**Additional file 1: Table S1.** Distribution of chronic illness by chronic illness with and without at least one disability - 2016. **Table S2.** Currently enrolled or received any assistant from any Social Safety Net Program (SSNP) in the last 12 months among chronically ill people.

## Data Availability

All data relevant to this study has been included in the manuscript or uploaded as supplementary information. The source data, based on which analyses were performed, cannot be shared publicly by the authors because of the data sharing restriction of Bangladesh Bureau of Statistics. However, the restricted source data are available from the Bangladesh Bureau of Statistics Institutional Data Access / Ethics Committee (contact via Bangladesh Bureau of Statistics, Computer Wing, E-27/A, Parishankhyan Bhaban, Agargaon, Dhaka, Bangladesh; website: http://data.bbs.gov.bd/index.php/catalog/HIES or http://data.bbs.gov.bd/index.php/catalog/182) on special request for researchers who meet the criteria for access to confidential data. The preliminary and final reports of the analyses of HIES2016 data performed by the BBS are available on the BBS website: http://www.bbs.gov.bd/site/page/648dd9f5-067b-4bcc-ba38-45bfb9b12394/Income,-Expenditure-&-Poverty (accessed on 26 January 2022).

## Abbreviations

BBS: Bangladesh Bureau of Statistics
BDT: Bangladeshi taka
CHE: Current health expenditure
CI: Confidence interval
GDP: Gross domestic product
GEC: Global Environmental Change
HIES: Household income and expenditure survey
ICF: International classification of functioning
ICFDH: ICF, Disability and Health
IPCC: Intergovernmental panel on climate change
LMIC: Low and middle-income country
NCD: Non-communicable disease
NGO: Non-government organization
NHP: National health policy
OOP: Out-of-pocket
OOPE: Out-of-pocket expenditure
OR: Odds ratio
PC: Principal component
PSU: Primary sampling unit
SDG: Sustainable development goal
SSNP: Social safety net program
WHO: World Health Organization
