# Supplementary material for: Do the issues of religious minority and coastal climate crisis increase the burden of chronic illness in Bangladesh?
Source: BMC Public Health. 2022 Feb 10;22:270. doi: 10.1186/s12889-022-12656-5 (PMC8830131; doi:10.1186/s12889-022-12656-5)
Supplement: Supplementary file 1 — Additional file 1: Table S1. Distribution of chronic illness by chronic illness with and without at least one disability - 2016. Table S2. Currently enrolled or received any assistant from any Social Safety Net Program (SSNP) in the last 12 months among chronically ill people. [file 12889_2022_12656_MOESM1_ESM.docx]

**Article title:** Do the issues of religious minority and coastal climate crisis enhance the burden of chronic illness in Bangladesh?

**Journal name:** BMC Public Health

**Author names:** Altaf Hossain^1*^, Md. Jahangir Alam^2*^, Janardhan Mydam^3, 4^ and Mohammad Tareque^5^

^1^Department of Statistics, Islamic University, Kushtia 7003, Bangladesh

^2^Department of Statistics, University of Rajshahi, Rajshahi 6205, Bangladesh

^3^Division of Neonatology, Department of Pediatrics, John H. Stroger, Jr. Hospital of Cook County, 1969 Ogden Avenue, Chicago, IL 60612, USA

^4^Department of Pediatrics, Rush medical center, Chicago, USA

^5^Bangladesh Institute of Governance and Management, Dhaka, Bangladesh

* Corresponding Author

**Affiliation and e-mail of the corresponding authors:**

Md. Jahangir Alam (MJA)

Department of Statistics, University of Rajshahi, Rajshahi 6205, Bangladesh

E-mail: jahangir_statru63@yahoo.com

Altaf Hossain (AH)

Department of Statistics, Islamic University, Kushtia 7003, Bangladesh

E-mail: rasel_stat71@yahoo.com

**Table S1.** Distribution of chronic illness by chronic illness with and without at least one disability - 2016

| Chronic illness | **Overall**  ***n*** (%) | Chronic illness with | |
| --- | --- | --- | --- |
|  |  | At least one disability  *n* (%) | **No disability**  *n* (%) |
| Chronic fever | 1330 (4.06) | 113 (8.50) | 1217 (91.50) |
| Injuries/Disability | 1567 (4.78) | 397 (25.34) | 1170 (74.66) |
| Chronic heart disease | 2441 (7.45) | 435 (17.82) | 2006 (82.18) |
| Respiratory Diseases/Asthma/Bronchitis | 3351 (10.23) | 636 (18.98) | 2715 (81.02) |
| Diarrhoea/Dysentery | 360 (1.10) | 52 (14.44) | 308 (85.56) |
| Gastric or ulcer | 6704 (20.46) | 815 (12.16) | 5889 (87.84) |
| Blood pressure | 3170 (9.67) | 531 (16.75) | 2639 (83.25) |
| Arthritis/Rheumatism | 4522 (13.80) | 942 (20.83) | 3580 (79.17) |
| Skin problem | 929 (2.84) | 83 (8.93) | 846 (91.07) |
| Diabetes | 2235 (6.82) | 376 (16.82) | 1859 (83.18) |
| Cancer | 122 (0.37) | 34 (27.87) | 88 (72.13) |
| Kidney Diseases | 452 (1.38) | 58 (12.83) | 394 (87.17) |
| Liver Diseases | 282 (0.86) | 32 (11.35) | 250 (88.65) |
| Mental Health | 522 (1.59) | 257 (49.23) | 265 (50.77) |
| Paralysis | 447 (1.36) | 262 (58.61) | 185 (41.39) |
| Ear/ENT problem | 734 (2.24) | 170 (23.16) | 564 (76.84) |
| Eye problem | 969 (2.96) | 400 (41.28) | 569 (58.72) |
| Others | 2631 (8.03) | 341 (12.96) | 2290 (87.04) |
| Total | 32,768 (100.0) | 5,934 (18.11) | 26,834 (81.89) |

**Table S2.** Currently enrolled or received any assistant from any Social Safety Net Program (SSNP) in the last 12 months among chronically ill people

|  |  | **Chronic illness with** | |
| --- | --- | --- | --- |
| Characteristic | **Overall**  ***n* (%)** | At least one  disability  *n* (%) | **No**  **disability**  *n* (%) |
| Currently enrolled/received any assistance from any SSNP |  |  |  |
| Yes | 3539 (10.80) | 1107 (18.66) | 2432 (9.06) |
| No | 29229 (89.20) | 4827 (81.34) | 24402 (90.94) |
| In which program has included in the last 12 months? |  |  |  |
| Ananda School (ROSC) [Cash/kind] | 45 (1.27) | 12 (1.08) | 33 (1.36) |
| Stipend for Primary Students | 153 (4.32) | 13 (1.17) | 140 (5.76) |
| School Feeding Program | 13 (0.37) | 1 (0.09) | 12 (0.49) |
| Stipend for Secondary and higher students | 135 (3.81) | 10 (0.90) | 125 (5.14) |
| Stipend for Dropout Students | 4 (0.11) | 0 (0.00) | 4 (0.16) |
| Stipend for Disabled Students | 24 (0.68) | 20 (1.81) | 4 (0.16) |
| Old Age Allowance | 1280 (36.17) | 577 (52.12) | 703 (28.91) |
| Widow/Deserted/Destitute Women Allowances | 309 (8.73) | 99 (8.94) | 210 (8.63) |
| MAP for the Poor Lactating Mothers | 8 (0.23) | 1 (0.09) | 7 (0.29) |
| Maternal Health Voucher Scheme | 1 (0.03) | 0 (0.00) | 1 (0.04) |
| Honorarium for Insolvent Freedom Fighters | 47 (1.33) | 15 (1.36) | 32 (1.32) |
| HMA for Injured Freedom Fighters | 19 (0.54) | 6 (0.54) | 13 (0.53) |
| Ration for Shaheed Family and Injured Freedom Fighters | 4 (0.11) | 0 (0.00) | 4 (0.16) |
| Allowances for Distressed Cultural Personalities/Activists | 1 (0.03) | 0 (0.00) | 1 (0.04) |
| Allowances for the Financially Insolvent Disabled | 102 (2.88) | 84 (7.59) | 18 (0.74) |
| Vulnerable Group Development (VGD) | 110 (3.11) | 19 (1.72) | 91 (3.74) |
| Vulnerable Group Feeding (VGF) | 461 (13.03) | 103 (9.30) | 358 (14.72) |
| General Relief Activities | 46 (1.30) | 11 (0.99) | 35 (1.44) |
| Gratuitous Relief (GR)- Food/ Cash | 381 (10.77) | 54 (4.88) | 327 (13.45) |
| Allowance for Beneficiaries in CTG-Hill Tracts Area | 6 (0.17) | 1 (0.09) | 5 (0.21) |
| Food Assistance in CTG-Hill Tracts Area | 18 (0.51) | 3 (0.27) | 15 (0.62) |
| Employment Generation Programme for the Ultra Poor | 17 (0.48) | 1 (0.09) | 16 (0.66) |
| Food/Cash For Work (FFW/CFW) | 13 (0.37) | 4 (0.36) | 9 (0.37) |
| Test Relief (TR) Food (cash) | 175 (4.94) | 44 (3.97) | 131 (5.39) |
| RERMP | 1 (0.03) | 0 (0.00) | 1 (0.04) |
| One Household One Farm | 2 (0.06) | 0 (0.00) | 2 (0.08) |
| Housing Support | 15 (0.42) | 1 (0.09) | 14 (0.58) |
| Agriculture Rehabilitation | 2 (0.06) | 0 (0.00) | 2 (0.08) |
| Targeted Ultra Poor (TUP) (BRAC) | 5 (0.14) | 0 (0.00) | 5 (0.21) |
| Char Livelihood Project | 1 (0.03) | 0 (0.00) | 1 (0.04) |
| Economic Empowerment for the Poor/Shiree | 3 (0.08) | 2 (0.18) | 1 (0.04) |
| Urban Partnership for Poverty Reduction (UPPR) | 2 (0.06) | 1 (0.09) | 1 (0.04) |
| Shouhardo Program | 1 (0.03) | 1 (0.09) | 0 (0.00) |
| Others | 135 (3.81) | 24 (2.17) | 111 (4.56) |
| How had you been selected in the program? |  |  |  |
| Applied and selected | 483 (13.65) | 171 (15.45) | 312 (12.83) |
| Persued/someone referred and selected | 1376 (38.88) | 454 (41.01) | 922 (37.91) |
| Selection committee selected | 1532 (43.29) | 439 (39.66) | 1093 (44.94) |
| NGO selected | 20 (0.57) | 6 (0.54) | 14 (0.58) |
| Other (specify) | 8 (0.23) | 2 (0.18) | 6 (0.25) |
| Don’t know | 120 (3.39) | 35 (3.16) | 85 (3.49) |
| Paid any money to be included in SSNP |  |  |  |
| Yes | 278 (7.86) | 112 (10.12) | 166 (6.83) |
| No | 3261 (92.14) | 995 (89.88) | 2266 (93.17) |

MAP: Maternity Allowance Programme, HMA: Honorarium & Medical Allowances, RERMP: Rural Employment and Road Maintenance Programme.
